# Leishmaniose viscerale et leucemie aigüe lymphoblastique B: quel est le rapport?

**DOI:** 10.11604/pamj.2015.20.53.6013

**Published:** 2015-01-20

**Authors:** Hind El Youssi, Aziz Touaoussa, Imane Bergui, Nawal Bougrine, Moncef Hassani Amrani

**Affiliations:** 1 Laboratoire d'Hématologie CHU Hassan II, Fès, Maroc

**Keywords:** Leishmaniose viscerale, leucemie aigüe lymphoblastique, traitement, visceral leishmaniasis, acute lymphoblastic leukemia, treatment

## Abstract

L'association leishmaniose viscérale et leucémie aigue a été rarement rapportée dans la littérature, cependant le diagnostic concomitant de ces deux entités n'a jamais été rapporté au Maroc. Le lien entre ces deux pathologies n'a pas encore été établi et le traitement n'a pas encore été codifié. Nous rapportons le cas d'un garçon de 12 ans chez qui une leishmaniose viscérale et une leucémie aigue lymphoblastique type B ont été diagnostiquées simultanément. Malgré l'administration d'un traitement antiparasitaire associé à une chimiothérapie l’évolution était marquée par le décès du patient.

## Introduction

Au Maroc la leishmaniose viscérale constitue une anthropozoonose endémique non contrôlée et sévit essentiellement sous sa forme méditerranéenne infantile. Elle est causée par l'espèce leishmania infantum. Cette parasitose connait depuis quelques années une recrudescence importante au Maroc, elle est rencontrée de façon habituelle chez l'enfant en rapport avec l'immaturité de ces moyens de défense immunitaire. Les rares cas retrouvés chez l'adulte surviennent généralement dans un contexte d'immunodépression. De nombreux mécanismes physiopathologiques sont incriminés dans la genèse des leucémies aigues lymphoblastiques de l'enfant et le rôle oncogène des leishmanies a été évoqué. Nous rapportons une observation qui soulève ce problème.

## Patient et observation

C'est un enfant âgé de 12 ans; sans antécédents particuliers qui a consulté en pédiatrie pour asthénie plus pâleur évoluant depuis 3 mois, l'examen clinique a objectivé une splénomégalie à 3 TDD avec polyadénopathies centimétriques cervicales, sous axillaires et inguinales. A la biologie: Anémie normochrome normocytaire arégénérative à 5 .8 g/dl d'hémoglobine. Une hyperleucocytose à 31610 éléments /mm3 avec PNN à 7400 éléments /mm3.Une thrombopénie à 28000 éléments /mm3. CRP: 24 mg/l; LDH: 598 UI/l; Le frottis du sang périphérique a mis en évidence la présence de 20% de blastes indifférenciés.

**Figure 1:**
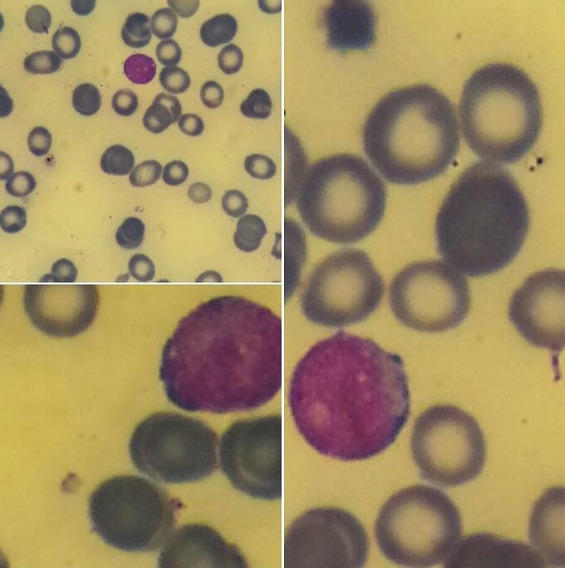
Myélogramme: présence de blastes et de corps de leishmanies dans leur forme amastigote « MGG, ×100 »

Le myélogramme a objectivé une moelle hypercellulaire hétérogène infiltrée par une population blastique estimée à 57%; il s'agit de cellule de taille variable, noyau souvent arrondi, chromatine fine et nucléolée, cytoplasme généralement peu abondant et agranulé, dont la coloration à la myélopéroxydase est négative. Par ailleurs, on a noté la présence de nombreux corps de leishmanies en intra et extra cellulaire sous la forme amastigote. En revanche les lignées granuleuses et erythroblastique sont hypoplasiques (Figure 1). L'immunophénotypage sur sang médullaire a montré une population blastique CD45 avec positivité des marqueurs lymphoïdes B CD 19, CD79a, CD20, CD22, IGM et de la TDT en faveur d'une leucémie aigue lymphoblastique B. La sérologie de la leishmaniose par immunofluorescence indirecte était positive. Par ailleurs les sérologies VIH, HVB, HVC étaient négatives. L’échographie abdominale a révélé une volumineuse splénomégalie homogène. Le diagnostic d'une leucémie aigue lymphoblastique B associé à une leishmaniose viscérale a été retenu.

Un traitement antiparasitaire à base du glucantime a été démarré en premier, à j 3 du traitement le patient a été mis sous chimiothérapie en phase d'induction. A J8 du traitement antiparasitaire le patient a présenté une thrombopénie profonde contre indiquant les injections IM du glucantime. L'indication de l'amphotéricine B a été posé mais le patient n'avait pas les moyens d'avoir le médicament. Le patient a été transfusé par des culots plaquettaires permettant la réintroduction du glucantime. A j36 de la phase d'induction, la chimiothérapie a été arrêtée devant l'installation d'un syndrome infectieux, hémorragique et anémique. Le médullogrammme de contrôle était en faveur d'une aplasie médullaire post chimiothérapie avec persistance de quelques corps de leishmanie.

La radiographie pulmonaire a objectivé un foyer basipulmonaire prenant la presque totalité du poumon gauche. L'enfant a été mis sous plusieurs associations d'antibiotiques à large spectre associés au facteur de croissance et des transfusions de culots plaquettaires, globulaires et plasma frais congelé. L’évolution a été marquée par l'installation d'un état de choc septique conduisant au décès du patient.

## Discussion

L'observation de ce patient soulève la question d'un lien possible entre la leishmaniose et le déclenchement d'une leucémie aigue lymphoblastique chez l'enfant. Cette association a été rarement rapportée dans la littérature; En 1983 un cas de leishmaniose viscérale en post chimiothérapie d'une leucémie aigue chez un adolescent de 15 ans a été rapporté par J.M.Aguado en Espagne [1]; un cas similaire a été rapporté en Iran par Fakhar M chez une fille de 12 ans [2]. Par ailleurs en 2011 un cas de LAL B survenant après traitement d'une leishmaniose viscérale chez un adulte de 20 ans a été rapporté par H Nafil à Marrakech [3]. Dans une revue de la littérature s'intéressant à l'association leishmaniose et affection maligne, Kopterides et al ont pu identifier globalement quatre situations cliniques [4]: 1) une leishmaniose avec une présentation déroutante mimant une tumeur (cutanée muqueuse ou organique) souvent responsable d'un retard diagnostique et thérapeutique; 2) la survenue d'une leishmaniose comme infection opportuniste dans les suites précoces ou tardives d'un traitement d'un cancer solide ou d'une hémopathie; 3) le diagnostic concomitant des deux entités par la coexistence des parasites et des cellules néoplasiques dans un même tissus ou organe (ganglions lymphatiques, moelle osseuse, foie); 4) la dégénérescence maligne d'une localisation cutanée ou viscérale d'une leishmaniose des mois voir des années après le diagnostic de celle-ci avec un probable rôle oncogène direct ou indirect des parasites.

Notre cas fait partie de la 3ème situation, où la leishmaniose et la LAL B ont été diagnostiquées simultanément sur le même myélogramme. S'agit il d'une simple coïncidence ou existe il un vrai lien de causalité entre ces deux pathologies? S'agit-il d'une immunodépression accrue due à la LAL B favorisant la survenue d'une leishmaniose comme infection opportuniste? Depuis 1979, la leishmaniose viscérale est connue comme infection survenant chez les patients immunodéprimés dont l'immunodépression est liée à une cause autre que l'infection à VIH [5]. Sa survenue chez des patients soumis a une thérapeutique immunosuppressive lourde et prolongée ainsi que des sujets fortement débilités (pathologie néoplasique, insuffisance rénale chronique, malnutrition...) vient confirmer le caractère opportuniste de cette parasitose. Les pathologies favorisant la leishmaniose viscérales en toutes en commun des déficits profonds de production en interféron gamma (INF gamma) par les lymphocytes T induisant, une baisse de l'activité macrophagique résultant du processus pathologique lui-même ou l'emploi de produits immunosuppresseurs, les principales étiologies sont les suivantes: LAL, LMC, MH, lupus ED, crohn, sarcoïdose, et fièvre typhoïde [6]. S'agit-il d'une dégénérescence maligne d'une leishmaniose viscérale? En effet une dégénérescence maligne de certaines localisations cutanées ou viscérales d'une leishmaniose a été rapportée [7]. Aussi la découverte d'importante dysplasie avec mitoses anormales ainsi qu'un infiltrat diffus essentiellement de type B sur des biopsies de lésions cutanées infectées par ces parasites seraient en faveur de cette hypothèse [8, 9]. cette effet serait multifactoriel et passe essentiellement par une aggravation de l’état d'immunodépression freinant d'avantage l'immunité anti tumoral suite a l'infestation par les leishmanies des cellules de la lignée blanche et l'altération de l'activation et du fonctionnement des macrophages et des cellules dendritiques [10]. L'instauration par le parasite d'un état inflammatoire chronique favorisant l'induction et/ou la promotion des néoplasies ou augmentant la susceptibilité individuelle a certains carcinogènes seraient également en cause [4]. Les options thérapeutiques d'une leishmaniose viscérale déjà limitées sont encore plus restreinte chez les sujets atteints d'une leucémie, cela est du au terrain fragilisé et aux nombreuses interactions avec les médicaments immunosuppresseurs.

Depuis le développement des formulations lipidiques de l'amphotéricine B dans les années 1990, un regain d'intérêt de ce médicament est noté. En effet, avec une efficacité comparable a l'AMB, l'AMB liposomale est peu néphrotoxique ainsi ce médicament est actuellement recommandé comme traitement de 1ère ligne de la leishmaniose viscérale mais son cout élevé reste la principale limitation de son utilisation à large échelle, ainsi les antimoniés pentavalent demeurent le traitement de référence dans notre pays vue le manque de moyens de la population traitée même si leur utilisation est souvent associé a de multiples effets indésirables. Chez notre malade l'AMB a été indiquée comme traitement de 2ème intention après l'installation d'une thrombopénie contre indiquant les injections du glucantime mais vu le manque de moyens du patient, il n'a pas pu en bénéficier. Cependant le traitement de l'association leishmaniose et LAL B n'est pas codifié; en effet il n'existe actuellement aucune étude évaluant un schéma thérapeutique pour cette association. Des études prospectives seraient utiles pour définir les modalités d'un consensus thérapeutique bien établi et démontrer son efficacité.

## Conclusion

Notre observation est originale par l'association d'une LV et d'une LAL B chez un enfant. La complexité du diagnostic et de la prise en charge concomitante de deux pathologies participent également à la singularité de cette observation. Des études doivent être menées dans le futur à fin d’élucider si cette rare association était une simple coexistence fortuite de deux troubles ou l'un a incité l'autre.
